# Retinal microcirculatory and choroidal structural characteristics of retinal vein occlusion-related macular edema with serous retinal detachment

**DOI:** 10.3389/fmed.2026.1941751

**Published:** 2026-10-07

**Authors:** Xianxian Kong, Fengjiao Li, Yalin Wang, Jing Wang, Weiyan Zhou

**Affiliations:** Department of Ophthalmology, Shandong Provincial Hospital Affiliated to Shandong First Medical University, Jinan, Shandong, China

**Keywords:** choroidal vascularity index, macular edema, optical coherence tomography angiography, retinal microcirculation, retinal vein occlusion, serous retinal detachment

## Abstract

**Objective:**

To characterize retinal microcirculation and choroidal structure in treatment-naive retinal vein occlusion-related macular edema (RVO-ME) eyes with/without serous retinal detachment (SRD) using ultra-widefield swept-source OCT angiography (UWF-SS-OCTA).

**Methods:**

This retrospective single-center study included 69 treatment-naive RVO-ME eyes (35 CRVO and 34 BRVO) classified by OCT-defined SRD. Between-group comparisons included CMT, FAZ metrics, sectoral VFD of the SCP, MCP, and DCP, SFCT, and three-dimensional choroidal metrics (CVV, CSV, CVI, and CSI). The location and extent of the occluded retinal region were also analyzed in BRVO eyes.

**Results:**

Thirty-five CRVO and 34 BRVO eyes were included. In both subtypes, SRD eyes had shorter disease duration, greater CMT (CRVO: 795.19 ± 240.83 vs. 397.84 ± 136.56 μm; BRVO: 633.53 ± 214.39 vs. 366.47 ± 86.20 μm), and lower FAZ circularity index (CRVO: 0.64 ± 0.23 vs. 0.84 ± 0.06; BRVO: 0.80 ± 0.07 vs. 0.86 ± 0.08) than non-SRD eyes (all *P* < 0.05). CRVO-SRD eyes had worse BCVA and a higher proportion of ischemic eyes (13/16 vs. 6/19) than non-SRD eyes (both *P* < 0.05). In BRVO, occlusion location was similar between groups (superotemporal branch most common), but SRD eyes had more extensive retinal involvement (*P* < 0.05). BRVO-SRD eyes showed reduced retinal vascular flow density in most macular sectors. In CRVO-SRD eyes, middle capillary plexus flow density was consistently reduced across all sectors. BRVO-SRD eyes showed greater SFCT, CSV, and CVV, whereas CRVO-SRD eyes showed higher foveal CSI and lower CVI in selected sectors.

**Conclusions:**

In treatment-naive RVO-ME eyes evaluated by UWF-SS-OCTA, SRD was associated with greater macular thickening, more irregular FAZ morphology, and more pronounced retinal microcirculatory impairment, with MCP-VFD reduction as the most consistent feature. Choroidal volume expansion in BRVO and compositional remodeling in CRVO suggest subtype-specific choroidal involvement; together, these findings support the potential value of layer-specific retinal perfusion metrics and three-dimensional choroidal parameters for imaging-based stratification.

## 1 Background

Globally, retinal vein occlusion (RVO) affects approximately 5.2 per 1,000 individuals and is recognized as a common cause of visual impairment, ranking second only to diabetic retinopathy among retinal vascular diseases (1). Macular edema is a common complication of RVO and represents the leading cause of visual impairment in patients with RVO (2).

Optical coherence tomography (OCT), a first-line modality for diagnosing RVO-ME, has demonstrated that RVO-ME can present with various morphological features, including sponge-like diffuse retinal thickening, cystoid macular edema (CME), and SRD (3). Previous studies have suggested that SRD is not merely an exudative manifestation; its occurrence often indicates more pronounced impairment of the outer blood-retinal barrier, particularly retinal pigment epithelium function, and its development may be associated with elevated inflammatory factor levels, altered choroidal hemodynamics, and disruption of specific microstructures, such as loss of external limiting membrane integrity (4).

In addition to ischemia and leakage within the retina itself, the choroid may also play an important role in the occurrence and progression of RVO-ME. The choroid is not only an important source of blood supply for the outer retina and the retinal pigment epithelium, but also a key structure for maintaining metabolic homeostasis and fluid transport balance in the macular region (5). Venous stasis, local hypoxia, and inflammatory cascades caused by RVO may be accompanied by changes in choroidal blood perfusion and vascular structure (6–8). Accordingly, investigating choroidal structural biomarkers, including the choroidal vascularity index (CVI), choroidal vascular volume (CVV), choroidal stromal index (CSI), and choroidal stromal volume (CSV), may provide deeper insights into choroidal vascular and stromal changes and their associations with SRD subtypes.

Over recent years, optical coherence tomography angiography (OCTA) has become a noninvasive, rapid, and reproducible three-dimensional imaging modality for the high-resolution quantitative assessment of retinal and choroidal microvascular architecture. By enabling quantitative analyses of parameters such as vessel density and blood flow perfusion, OCTA compensates for some limitations of fluorescein angiography (9). Among available systems, UWF-SS-OCTA further expands the observable area and improves visualization of both retinal and choroidal circulation, making it particularly suitable for assessing vascular alterations in RVO (10).

Although studies applying OCTA to RVO have gradually increased, important knowledge gaps remain. First, most existing studies have focused on overall vascular alterations in RVO or analyses of single parameters, and systematic comparative studies of multidimensional retinal and choroidal biomarkers between RVO-ME subtypes with and without SRD are lacking (11, 12). Second, evidence regarding the associations between choroidal structural metrics and SRD remains limited. Group differences in retinal vessel density and foveal avascular zone (FAZ)-related parameters under different SRD statuses also remain unclear (13). In addition, previous studies have mostly analyzed only the superficial capillary plexus (SCP) and deep capillary plexus (DCP), or have combined the middle capillary plexus (MCP) with the DCP as a “deep vascular complex (DVC)” (14, 15), whereas relatively little attention has been paid to the MCP alone (16, 17). However, as an important intermediate layer connecting the SCP and DCP, the MCP is relatively independent in terms of vascular composition and drainage patterns (16); if it is combined with the DCP, layer differences may be obscured. Therefore, evaluating MCP vessel density helps to identify in greater detail the layer-specific microcirculatory changes that may exist in different RVO-ME subtypes.

Against this background, this study aimed to use UWF-SS-OCTA to compare ocular morphological and microcirculatory characteristics in patients with RVO-ME with and without SRD. By systematically collecting and comparing intergroup differences in central macular thickness (CMT), subfoveal choroidal thickness (SFCT), vessel densities of the SCP, MCP, DCP, FAZ-related parameters, and more refined three-dimensional choroidal structural parameters (CVI, CVV, CSI, and CSV) across groups, we sought to explore the association between the SRD subtype and retinal microcirculation as well as choroidal imaging features, thereby providing imaging evidence for subsequent studies.

## 2 Methods

### 2.1 Patients

This retrospective comparative single-center study was approved by the review board of Shandong Provincial Hospital Affiliated to Shandong First Medical University. We retrospectively reviewed the medical records of patients diagnosed with RVO, including BRVO and CRVO, at Shandong Provincial Hospital Affiliated to Shandong First Medical University between February 2025 and May 2026. This study was conducted in accordance with the Declaration of Helsinki, and written informed consent was obtained from all patients. We screened 69 eyes from 69 patients with unilateral RVO-ME, and all included eyes were treatment-naive at the time of imaging, with no prior intravitreal anti-VEGF injections, intravitreal or periocular glucocorticoid therapy, retinal laser photocoagulation, or vitreoretinal surgery.

Each study eye had UWF-SS-OCTA imaging with the TowardPi system and underwent a comprehensive ophthalmic examination, including fluorescein fundus angiography (FFA), best-corrected visual acuity (BCVA, expressed as logMAR), slit-lamp biomicroscopy, and dilated fundus examination. Baseline clinical data were also collected from the medical records, including age, sex, affected eye, type of RVO, disease duration (time from symptom onset to first imaging), BCVA, spherical equivalent (SE), retinal ischemic status on FFA (ischemic/non-ischemic), systemic vascular risk factors such as hypertension, diabetes, and hyperlipidemia, as well as the OCTA image signal strength index.

For eyes with BRVO, the location of the occluded vessel was recorded according to FFA and OCTA and classified as superotemporal, inferotemporal, superonasal, or inferonasal branch occlusion; eyes in which the occluded region spanned two or more branch territories within the same hemiretina (for example, combined superotemporal and superonasal involvement) were classified as extensive hemispheric involvement (denoted as “superior, extensive” or “inferior, extensive” in Table 2). The extent of the involved retinal area was expressed as the proportion of the involved area to the total retinal area and categorized by quadrant (for example, an involved area corresponding to one quarter of the retina was classified as one quadrant).

RVO cases were selected according to the following criteria: (1) CRVO or BRVO confirmed by fundus examination, fundus fluorescein angiography (FFA), and OCTA; (2) macular edema confirmed by OCTA, defined as CMT > 250 μm; (3) spherical equivalent between −6.0 and +6.0 D; and (4) OCTA signal strength index greater than 5, with no obvious motion artifacts or media opacity affecting image analysis. Cases were excluded if they had any of the following: (1) coexisting retinal diseases, including diabetic retinal disease, macular epiretinal membrane, or vitreomacular traction syndrome; (2) systemic conditions that could affect the fundus, including hematologic disorders, autoimmune diseases, neurodegenerative diseases, severe renal disease, or chronic renal failure; (3) other active ocular diseases, including uveitis or glaucoma, (4) prior RVO-related treatment before OCTA imaging; and (5) incomplete clinical or imaging data.

### 2.2 UWF-SS-OCTA image acquisition and analysis

The UWF-SS-OCTA device (TowardPi Medical Technology Co., Ltd, Beijing, China) uses a long-wavelength 1,060-nm laser and a scanning speed of 400,000 A-scans per second. In tissue, the axial resolution is approximately 3.8 μm, and the lateral resolution is estimated to be approximately 10 μm. The A-scan depth of this device in tissue is 6.0 mm (2,560 pixels). All images were acquired using a 24 mm × 20 mm scan pattern centered on the fovea, with 1,280 B-scan positions and 1,536 A-scans per B-scan.

According to the presence or absence of serous retinal detachment (SRD) on OCT images, patients with CRVO and those with BRVO were each divided into two groups. On OCT, SRD was morphologically defined as detachment of the neurosensory retina from the retinal pigment epithelium (RPE), with a uniform hyporeflective space between the two layers corresponding to subretinal fluid (18). CME was characterized on OCT by multiple cystoid hyporeflective spaces within the neuroepithelial layer.

The built-in software automatically segmented the retina and choroid into sublayers, and two senior ophthalmic imaging physicians independently performed manual verification of the automated segmentation results to correct segmentation errors, remove projection artifacts and motion artifacts, and then conduct parameter quantification after standardization. Both reviewers were blinded to the subjects' SRD status and clinical information. When disagreements arose, a consensus was reached through discussion; if disagreements persisted, the final decision was made by a third senior retinal specialist. Images from 20 randomly selected eyes were used, and two physicians independently measured the key imaging parameters, including CMT, FAZ area, and SFCT, without knowing each other's results. Inter-observer agreement was assessed using the intraclass correlation coefficient (ICC). An ICC < 0.50 indicated poor agreement, 0.50–0.75 indicated moderate agreement, 0.75–0.90 indicated good agreement, and >0.90 indicated excellent agreement.

The retinal sublayers included the SCP, MCP, and DCP. The choroidal vasculature was automatically stratified by vessel diameter into the choriocapillaris (CC) and the large and medium choroidal vessels (LMCV). The CC was defined as the zone within 29 μm beneath Bruch's membrane, whereas the LMCV compartment was defined as the zone extending from 29 μm beneath Bruch's membrane to the choroid-sclera interface.

CMT and SFCT were quantified on UWF-SS-OCTA B-scan images using the built-in caliper tool. The FAZ was automatically delineated on the full-retinal slab, which spanned from the internal limiting membrane to 6 μm beneath the outer plexiform layer. FAZ-related parameters included area (mm^2^), perimeter (mm), circularity index (CI), and flow density within a 300-μm-wide annular region surrounding the FAZ (FD-300, %). CI was calculated as CI = 4π × area/perimeter^2^, with values closer to 1 indicating a more regular FAZ shape. According to the ETDRS grid, vessel flow density (VFD, %) of the SCP, MCP, and DCP was measured separately in the foveal, superior (S), temporal (T), inferior (I), and nasal (N) regions using the built-in software. Choroidal three-dimensional (3D) parameters, including CVV (mm^3^), CVI, CSI, and CSV (mm^3^), were also measured. CVV represents the actual volume of choroidal vessels per unit area or unit volume. CVI is the ratio of choroidal vascular volume to total choroidal volume within a specified region. CSI is the ratio of choroidal stromal volume to total choroidal volume within a specified region. CSV represents the actual volume of choroidal stroma per unit area or unit volume.

### 2.3 Data analysis

All statistical analyses were performed using SPSS software (version 31.0; IBM Corp., Armonk, NY, USA). Continuous data are presented as the mean ± standard deviation (SD). The normality of each variable was examined using the Shapiro–Wilk test. When comparing the SRD and non-SRD groups within each RVO subtype (CRVO or BRVO), normally distributed data were analyzed using the independent-samples *t* test, and the mean difference (MD) and its 95% confidence interval (CI) were calculated. Non-normally distributed data were analyzed using the Mann–Whitney *U* test, and the median difference and its 95% CI were calculated based on the Hodges–Lehmann method. Categorical data (such as sex and affected eye) were evaluated with the chi-square test or Fisher's exact test, as appropriate. In eyes with BRVO, the distribution of occlusion locations between the two groups was compared using Fisher-Freeman-Halton exact test with Monte Carlo simulation (100,000 permutations). Inter-observer agreement for manual segmentation review was quantified using the two-way random-effects intraclass correlation coefficient (ICC, absolute agreement). Statistical significance was defined as two-sided *P* < 0.05.

## 3 Results

### 3.1 Comparison of general data

This study included 35 CRVO eyes (16 with SRD and 19 without SRD) and 34 BRVO eyes (19 with SRD and 15 without SRD). As shown in Table 1, age, sex, affected-eye laterality, spherical equivalent, OCTA signal strength index, and the prevalence of hypertension, diabetes mellitus, and hyperlipidemia did not differ significantly between the SRD and non-SRD groups among patients with either CRVO or BRVO (*P* > 0.05). In both RVO subtypes, disease duration, defined as the interval from symptom onset to the first imaging visit, was significantly shorter in the SRD group (CRVO: 11.88 ± 14.63 days; BRVO: 21.95 ± 16.51 days) than in the non-SRD group (CRVO: 32.63 ± 39.09 days; BRVO: 38.93 ± 26.47 days) [CRVO: mean difference(MD), −20.76 days; 95% CI, −40.76 to −0.75, *P* = 0.043; BRVO: MD, −16.99 days; 95% CI, −32.07 to −1.90; *P* = 0.029].

**Table 1:** Demographics profile and baseline ocular features of patients with RVO.

Characteristic	CRVO				BRVO			
	SRD group	Non-SRD group	Mean difference (95% CI)	*P*	SRD group	Non-SRD group	Mean difference (95% CI)	*P*
Number of eyes	16	19	–	–	19	15	–	–
Sex, male/female	10/6	10/9	–	0.557^a^	8/11	9/6	–	0.300^a^
Age (years)	62.19 ± 11.13	58.89 ± 15.07	3.29 (−5.97 to 12.56)	0.475^b^	54.84 ± 10.62	56.73 ± 8.29	−1.89 (−8.69 to 4.91)	0.575^b^
Disease eye, OD/OS	8/8	6/13	–	0.268^a^	9/10	4/11	–	0.217^a^
Disease duration (days)	11.88 ± 14.63	32.63 ± 39.09	−20.76 (−40.76 to −0.75)	**0** **.** **043** ^b^	21.95 ± 16.51	38.93 ± 26.47	−16.99 (−32.07 to −1.90)	**0** **.** **029** ^b^
Spherical equivalent (D)	0.95 ± 2.16	−0.28 ± 1.89	1.23 (−0.20 to 2.66)	0.089^b^	1.09 ± 1.44	0.61 ± 1.67	0.48 (−0.64 to 1.59)	0.387^b^
Hypertension, *n* (%)	9 (56.25)	10 (52.63)	–	1.000^a^	12 (63.16)	7 (46.67)	–	0.489^a^
Diabetes mellitus, *n* (%)	6 (37.50)	4 (21.05)	–	0.454^a^	4 (21.05)	3 (20.00)	–	1.000^a^
Hyperlipidemia, *n* (%)	5 (31.25)	4 (21.05)	–	0.700^a^	7 (36.84)	4 (26.67)	–	0.715^a^
Ischemic/non ischemic	13/3	6/13	–	**0** **.** **006** ^a^	13/6	5/10	–	0.082^a^
BCVA (logMAR)	0.99 ± 0.44	0.59 ± 0.65	0.40 (0.01 to 0.79)	0.043^b^	0.55 ± 0.33	0.44 ± 0.36	0.11 (−0.13 to 0.35)	0.359^b^
OCTA signal strength index	9.06 ± 0.85	9.37 ± 0.76	−0.31 (−0.86 to 0.28)	0.271^b^	9.13 ± 0.89	9.32 ± 0.82	−0.19 (−0.78 to 0.40)	0.513^b^

Data are presented as mean ± SD or number (percentage) of patients. BCVA, best-corrected visual acuity; BRVO, branch retinal vein occlusion; CRVO, central retinal vein occlusion; logMAR, logarithm of the minimum angle of resolution; OD, oculus dexter; OS, oculus sinister; SRD, serous retinal detachment. Significant *P* values are in bold.

a Fisher's exact test.

b Independent-sample *t*-test.

Among patients with CRVO, logMAR BCVA was significantly better in the non-SRD group (0.59 ± 0.65) than in the SRD group (0.99 ± 0.44) (MD, 0.40 logMAR; 95% CI, 0.01–0.79; *P* = 0.043), while the proportion of ischemic eyes was significantly higher in the SRD group (13/16) than in the non-SRD group (6/19) (*P* = 0.006). BCVA and proportion of ischemic eyes did not differ significantly between the two BRVO subgroups (*P* > 0.05) (see Table 1).

For BRVO, the occluded branch was most commonly superotemporal in both subgroups, and the distribution of occluded branch location did not differ significantly between SRD and non-SRD eyes (*P* > 0.05). However, the extent of the occluded retinal region was greater in the SRD group (2.19 ± 1.12 vs. 1.49 ± 0.82 quadrants; MD, 0.70; 95% CI, 0.07–1.33; *P* = 0.043) (see Table 2).

**Table 2:** Location and extent of the occluded retinal region in BRVO eyes by SRD status.

Parameter	SRD group	Non-SRD group	*P*
Location of the occluded branch, *n* (%)			0.548^a^
Superotemporal	15 (78.95)	13 (86.67)	
Inferotemporal	1 (5.26)	2 (13.33)	
Inferonasal	2 (10.53)	0 (0.00)	
Superior, extensive	1 (5.26)	0 (0.00)	
Extent of the occluded region (quadrants)	2.19 ± 1.12	1.49 ± 0.82	**0** **.** **043** ^b^

The extent of the occluded region was expressed as the proportion of the total retinal area involved, categorized in quadrants. “Superior, extensive” denotes occlusion involving both the superotemporal and superonasal branch territories (i.e., the entire superior hemiretina). SRD, serous retinal detachment. Significant *P* values are in bold.

a Fisher–Freeman–Halton exact test with Monte Carlo simulation (100,000 permutations).

b Independent-sample *t*-test.

### 3.2 Inter-observer agreement

The inter-observer agreement for CMT, FAZ area, and SFCT was excellent, with ICCs of 0.976 (95% CI, 0.94–0.99), 0.956 (95% CI, 0.89–0.98), and 0.974 (95% CI, 0.94–0.99) (*P* < 0.05) (see Table 3).

**Table 3:** Inter-observer agreement for CMT, FAZ area, and SFCT measurements.

Parameter	ICC (2,1)	95% CI	*P*
CMT (μm)	0.976	0.94–0.99	**<0** **.** **001**
FAZ area (mm^2^)	0.956	0.89–0.98	**<0** **.** **001**
SFCT (μm)	0.974	0.94–0.99	**<0** **.** **001**

ICC (2,1), intraclass correlation coefficient, two-way random-effects model, absolute agreement, single measurement; CI, confidence interval; CMT, central macular thickness; FAZ, foveal avascular zone; SFCT, subfoveal choroidal thickness. *n* = 20 eyes. Significant *P* values are in bold.

### 3.3 Retinal structural and microvascular parameters

#### 3.3.1 Central macular thickness and foveal avascular zone parameters

Figure 1 clearly illustrates the intergroup differences in CMT. Among patients with either CRVO or BRVO, CMT was significantly higher in the SRD group (CRVO: 795.19 ± 240.83 μm; BRVO: 633.53 ± 214.39 μm), than in the non-SRD group (CRVO: 397.84 ± 136.56 μm; BRVO: 366.47 ± 86.20 μm) (CRVO: MD, 397.35 μm; 95% CI, 265.40–529.30; *P* < 0.001; BRVO: MD, 267.06 μm; 95% CI, 155.82–378.29; *P* < 0.001) (see Table 4 and Figure 1).

**Figure 1:**
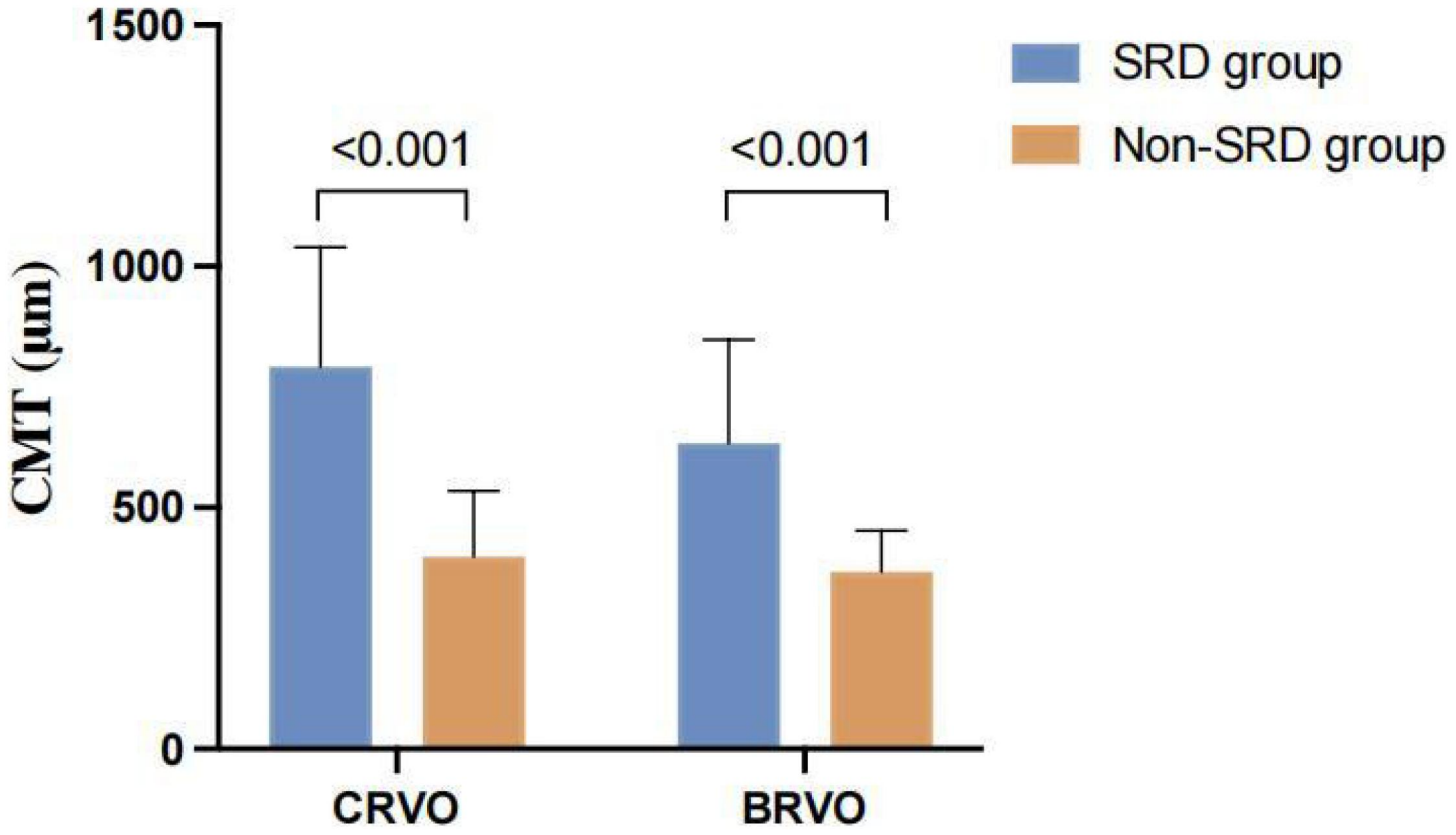
Comparison of CMT between SRD and non-SRD groups in CRVO and BRVO. Data were shown as mean ± SD, independent-sample *t*-test. *P* < 0.05 was considered statistically significant.

**Table 4:** Retinal structural and microvascular metrics by RVO subtype and SRD status.

Retinal parameter	CRVO				BRVO			
	SRD group	Non-SRD group	Mean difference (95% CI)	*P* ^a^	SRD group	Non-SRD group	Mean difference (95% CI)	*P* ^a^
CMT (μm)	795.19 ± 240.83	397.84 ± 136.56	397.35 (265.40 to 529.30)	**<0** **.** **001**	633.53 ± 214.39	366.47 ± 86.20	267.06 (155.82 to 378.29)	**<0**.**001**
FAZ								
Area (mm^2^)	0.39 ± 0.19	0.39 ± 0.11	−0.001 (−0.11 to 0.11)	0.979	0.31 ± 0.13	0.32 ± 0.10	−0.01 (−0.09 to 0.08)	0.869
Perimeter (mm)	2.68 ± 0.80	2.41 ± 0.39	0.27 (−0.19 to 0.73)	0.233	2.16 ± 0.46	2.12 ± 0.32	0.04 (−0.27 to 0.32)	0.777
CI	0.64 ± 0.23	0.84 ± 0.06	−0.19 (−0.32 to −0.07)	**0** **.** **004**	0.80 ± 0.07	0.86 ± 0.08	−0.06 (−0.11 to −0.01)	**0** **.** **014**
FD-300 (%)	38.67 ± 18.20	42.04 ± 14.03	−3.37 (−14.46 to 7.71)	0.540	40.60 ± 12.17	46.41 ± 15.79	−5.81 (−15.57 to 3.95)	0.234
VFD (%)								
SCP								
Fovea	12.15 ± 10.85	18.54 ± 8.79	−6.39 (−13.14 to 0.36)	0.063	16.30 ± 8.36	29.67 ± 10.56	−13.36 (−20.21 to −6.52)	**<0** **.** **001**
Superior	36.03 ± 20.39	49.75 ± 19.69	−13.71 (−27.53 to 0.10)	0.052	32.15 ± 14.44	58.76 ± 16.34	−26.61 (−37.59 to −15.62)	**<0** **.** **001**
Temporal	30.85 ± 20.89	41.58 ± 17.49	−10.73 (−23.92 to 2.46)	0.107	33.89 ± 8.90	53.68 ± 11.80	−19.79 (−27.34 to −12.24)	**<0** **.** **001**
Inferior	38.48 ± 18.14	48.55 ± 18.00	−10.08 (−22.54 to 2.39)	0.110	44.29 ± 16.26	64.59 ± 18.55	−20.30 (−32.73 to −7.87)	**0** **.** **002**
Nasal	27.53 ± 21.18	48.14 ± 15.93	−20.61 (−33.38 to −7.84)	**0** **.** **002**	40.99 ± 13.76	61.01 ± 17.13	−20.02 (−30.80 to −9.24)	**<0** **.** **001**
MCP								
Fovea	7.42 ± 11.20	15.36 ± 10.62	−7.94 (−15.50 to −0.43)	**0** **.** **039**	7.93 ± 5.92	16.83 ± 10.08	−8.89 (−14.99 to −2.79)	**0** **.** **006**
Superior	15.12 ± 12.38	29.63 ± 11.34	−14.51 (−22.27 to −6.35)	**<0** **.** **001**	15.68 ± 11.84	27.85 ± 10.86	−12.17 (−20.61 to −4.21)	**0** **.** **004**
Temporal	18.11 ± 14.25	31.85 ± 12.14	−13.74 (−22.82 to −4.67)	**0** **.** **004**	22.79 ± 7.92	31.34 ± 10.86	−8.56 (−15.11 to −2.00)	**0** **.** **012**
Inferior	17.79 ± 11.48	30.47 ± 14.07	−12.68 (−21.62 to −3.74)	**0** **.** **007**	29.54 ± 12.88	36.77 ± 11.22	−7.22 (−15.79 to 1.35)	0.096
Nasal	15.64 ± 13.42	30.47 ± 10.70	−14.83 (−23.13 to −6.54)	**<0** **.** **001**	26.39 ± 9.71	36.70 ± 13.38	−10.32 (−18.38 to −2.26)	**0** **.** **014**
DCP								
Fovea	4.33 ± 3.67	4.36 ± 2.98	−0.03 (−2.32 to 2.25)	0.978	3.24 ± 3.15	5.12 ± 2.58	−1.88 (−3.93 to 0.17)	0.071
Superior	7.49 ± 6.12	10.21 ± 4.60	−2.71 (−6.40 to 0.97	0.144	6.06 ± 5.63	11.22 ± 6.52	−5.16 (−9.41 to −0.92)	**0** **.** **019**
Temporal	5.92 ± 4.73	8.11 ± 3.39	−2.19 (−4.99 to 0.61)	0.121	6.91 ± 3.83	10.95 ± 3.78	−4.03 (−6.87 to −1.36)	**0** **.** **004**
Inferior	6.75 ± 5.25	9.95 ± 3.40	−3.20 (−6.35 to −0.06)	**0** **.** **046**	9.22 ± 4.69	14.97 ± 9.38	−5.75 (−10.69 to −0.82)	**0** **.** **026**
Nasal	7.46 ± 5.75	9.70 ± 2.61	−2.23 (−5.48 to 1.01)	0.167	8.29 ± 4.01	12.76 ± 3.77	−4.47 (−7.22 to −1.73)	**0** **.** **002**

CMT, central macular thickness; FAZ, foveal avascular zone; CI, circularity index; FD-300, flow density in a 300-μm annulus surrounding the FAZ; VFD, vessel flow density; SCP, superficial capillary plexus; MCP, middle capillary plexus; DCP, deep capillary plexus. Significant *P* values are in bold.

a Independent-sample *t*-test.

Figure 2 shows changes in FAZ morphology. In both CRVO and BRVO patients, CI was significantly lower in the SRD group (CRVO: 0.64 ± 0.23; BRVO: 0.80 ± 0.07) than in the non-SRD group (CRVO: 0.84 ± 0.06; BRVO: 0.86 ± 0.08) (CRVO: MD, −0.19; 95% CI, −0.32 to −0.07; *P* = 0.004; BRVO: MD, −0.06; 95% CI, −0.11 to −0.01; *P* = 0.014), suggesting a more irregular FAZ configuration in RVO eyes with SRD. By contrast, FAZ area, perimeter, and FD-300 did not differ significantly between SRD and non-SRD groups in either subtype (*P* > 0.05) (see Table 4 and Figure 2).

**Figure 2:**
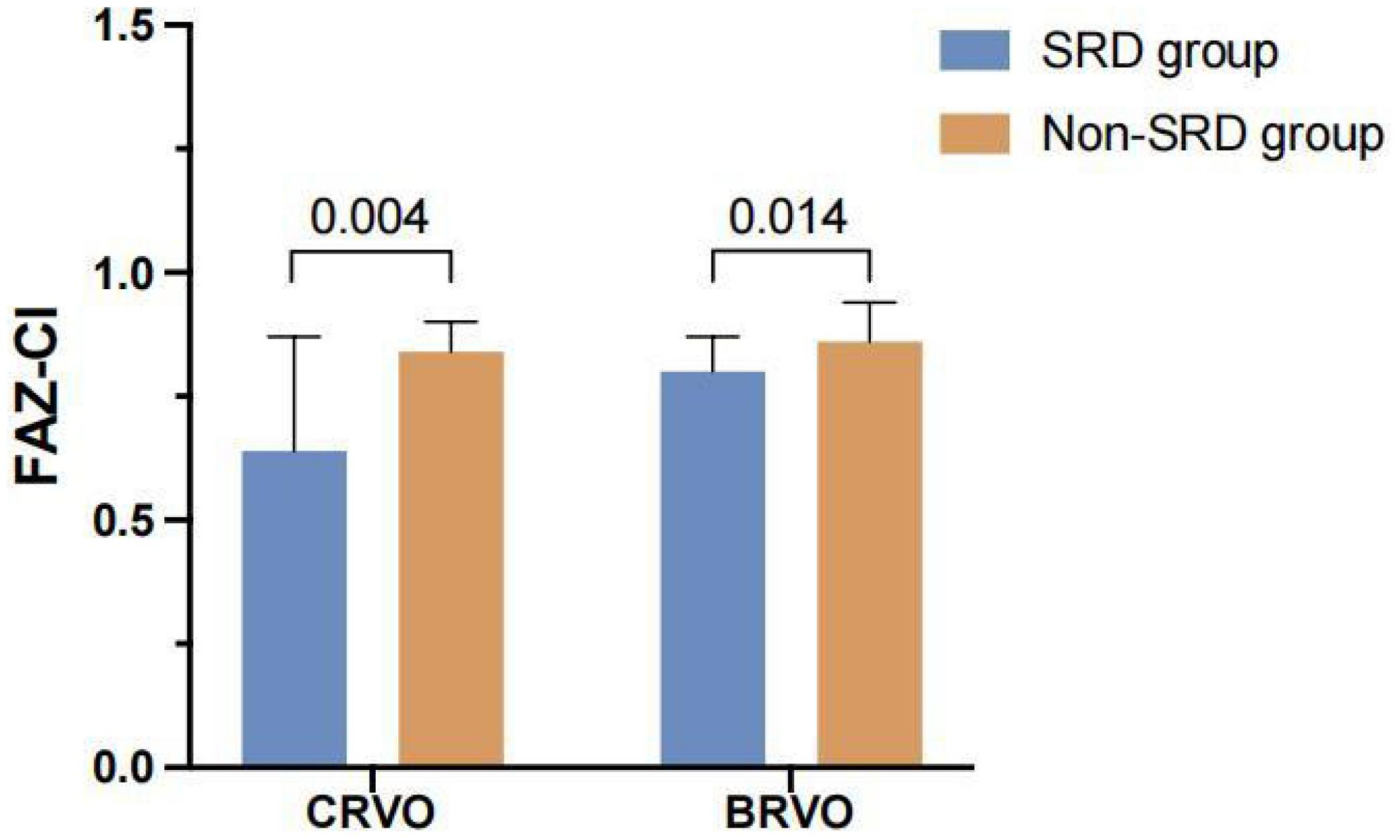
Comparison of FAZ-CI between SRD and non-SRD groups in CRVO and BRVO. Data were shown as mean ± SD, independent-sample *t*-test. *P* < 0.05 was considered statistically significant.

#### 3.3.2 Vessel flow density (VFD) in retinal plexuses

##### 3.3.2.1 Superficial capillary plexus (SCP)

As shown in Table 4, in CRVO patients, SCP-VFD reduction was limited to the nasal subfield, where SRD eyes (27.53 ± 21.18%) had lower values than non-SRD eyes (48.14 ± 15.93%) (MD, −20.61%; 95% CI, −33.38% to −7.84%; *P* = 0.002), whereas the foveal, superior, temporal, and inferior subfields showed no significant differences (*P* > 0.05). In BRVO, SRD was associated with a diffuse decrease in SCP-VFD across all macular subfields (fovea: 16.30 ± 8.36% vs. 29.67 ± 10.56%; superior: 32.15 ± 14.44% vs. 58.76 ± 16.34%; temporal: 33.89 ± 8.90% vs. 53.68 ± 11.80%; inferior: 44.29 ± 16.26% vs. 64.59 ± 18.55%; nasal: 40.99 ± 13.76% vs. 61.01 ± 17.13%) (*P* < 0.05) (see Table 4).

##### 3.3.2.2 Middle capillary plexus (MCP)

Figure 3 shows the intergroup differences in MCP-VFD among CRVO patients. In CRVO patients, MCP-VFD was significantly lower in the SRD group (fovea: 7.42 ± 11.20%; superior: 15.12 ± 12.38%; temporal: 18.11 ± 14.25%; inferior: 17.79 ± 11.48%; nasal: 15.64 ± 13.42%) than in the non-SRD group (fovea: 15.36 ± 10.62%; superior: 29.63 ± 11.34%; temporal: 31.85 ± 12.14%; inferior: 30.47 ± 14.07%; nasal: 30.47 ± 10.70%) (*P* < 0.05) (see Table 4 and Figure 3).

**Figure 3:**
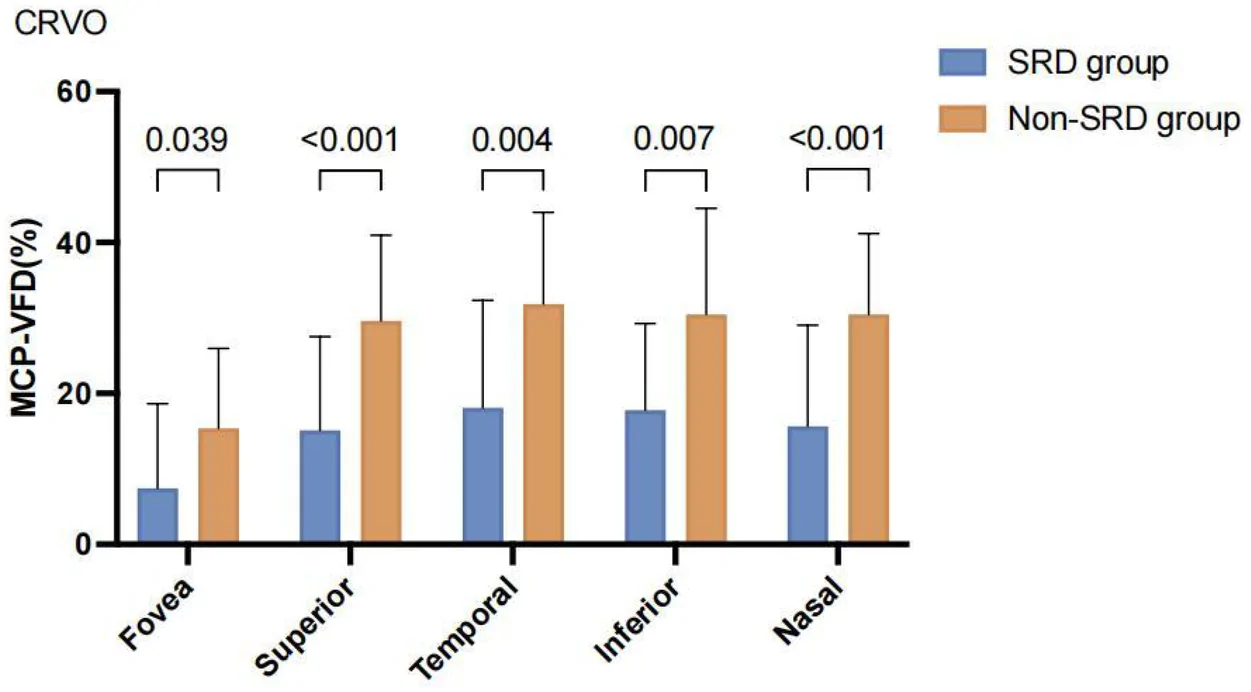
Comparison of MCP-VFD between SRD and non-SRD groups in CRVO. Data were shown as mean ± SD, independent-sample *t*-test. *P* < 0.05 was considered statistically significant.

Figure 4 shows the intergroup differences in MCP-VFD among BRVO patients. In BRVO patients, except for the inferior subfield, where no intergroup difference was detected (*P* > 0.05), MCP-VFD was significantly lower in the SRD group (fovea: 7.93 ± 5.92%; superior: 15.68 ± 11.84%; temporal: 22.79 ± 7.92%; nasal: 26.39 ± 9.71%) than in the non-SRD group (fovea: 16.83 ± 10.08%; superior: 27.85 ± 10.86%; temporal: 31.34 ± 10.86%; nasal: 36.70 ± 13.38%) in the remaining four macular subfields (*P* < 0.05) (see Table 4).

**Figure 4:**
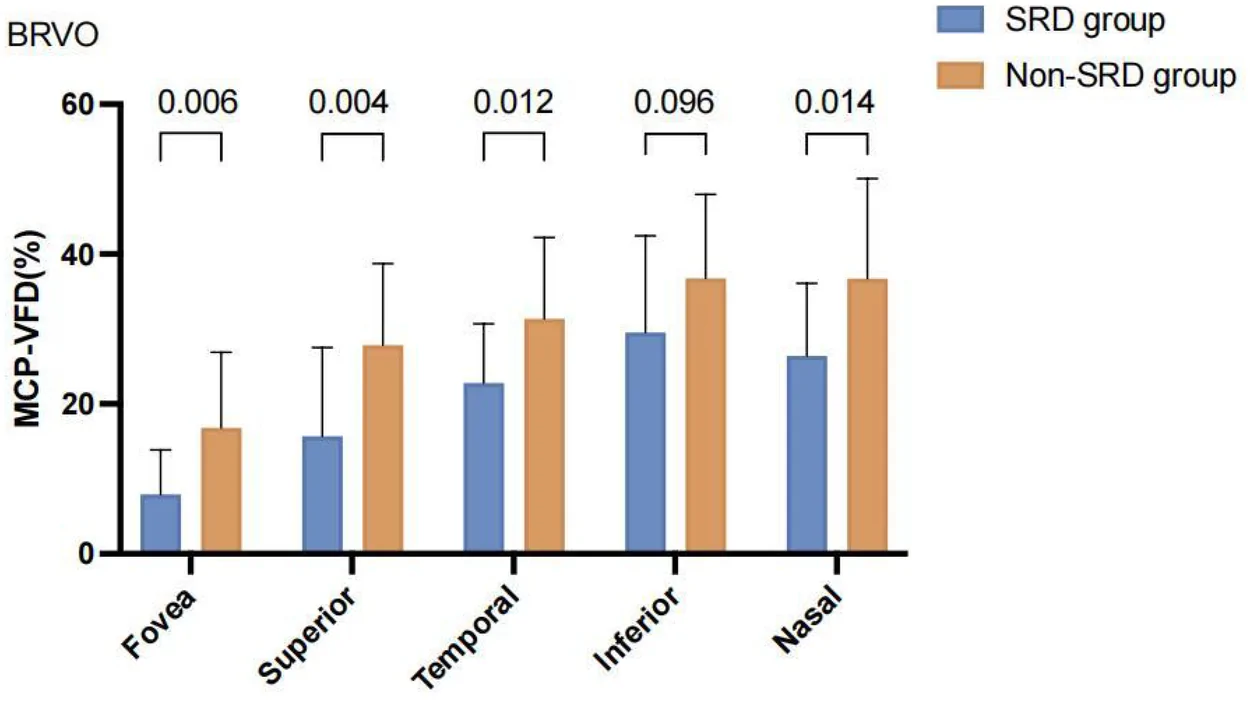
Comparison of MCP-VFD between SRD and non-SRD groups in BRVO. Data were shown as mean ± SD, independent-sample *t*-test. *P* < 0.05 was considered statistically significant.

##### 3.3.2.3 Deep capillary plexus (DCP)

In CRVO patients, DCP-VFD in the inferior region was significantly lower in the SRD group (6.75 ± 5.25%) than in the non-SRD group (9.95 ± 3.40%) (MD, −3.20%; 95% CI, −6.35% to −0.06%; *P* = 0.046). No intergroup differences were observed in the remaining subfields (*P* > 0.05) (see Table 4).

In BRVO patients, except for the foveal subfield, where the difference was not significant (*P* > 0.05), DCP-VFD was significantly lower in the SRD group (superior: 6.06 ± 5.63%; temporal: 6.91 ± 3.83%; inferior: 9.22 ± 4.69%; nasal: 8.29 ± 4.01%) than in the non-SRD group (superior: 11.22 ± 6.52%; temporal: 10.95 ± 3.78%; inferior: 14.97 ± 9.38%; nasal: 12.76 ± 3.77%) in the superior, temporal, inferior, and nasal subfields (*P* < 0.05) (see Table 4).

### 3.4 Subfoveal choroidal thickness and choroidal three-dimensional structural parameters

#### 3.4.1 Subfoveal choroidal thickness (SFCT)

No significant difference in SFCT was observed between the two groups among CRVO patients (*P* > 0.05). However, among BRVO patients, SFCT was significantly greater in the SRD group (413.74 ± 127.09 μm) than in the non-SRD group (309.53 ± 82.65 μm) (MD, 104.20 μm; 95% CI, 26.90–181.51; *P* = 0.010) (see Table 5).

**Table 5:** Choroidal structural parameters by RVO subtype and SRD status.

Choroidal parameter	CRVO				BRVO			
	SRD group	Non-SRD group	Mean difference (95% CI)	*P* ^a^	SRD group	Non-SRD group	Mean difference (95% CI)	*P* ^a^
SFCT (μm)	380.56 ± 151.87	332.68 ± 104.29	47.88 (−40.57 to 136.33)	0.279	413.74 ± 127.09	309.53 ± 82.65	104.20 (26.90 to 181.51)	**0** **.** **010**
CSV (mm^3^)								
Fovea	0.18 ± 0.07	0.15 ± 0.06	0.04 (−0.01 to 0.08)	0.094	0.19 ± 0.07	0.13 ± 0.06	0.06 (0.01 to 0.10)	**0** **.** **015**
Superior	0.30 ± 0.13	0.27 ± 0.10	0.03 (−0.05 to 0.10)	0.405	0.36 ± 0.14	0.26 ± 0.11	0.10 (0.01 to 0.19)	**0** **.** **037**
Temporal	0.33 ± 0.12	0.29 ± 0.12	0.04 (−0.04 to 0.11)	0.366	0.37 ± 0.14	0.28 ± 0.10	0.09 (0.01 to 0.18)	**0** **.** **031**
Inferior	0.29 ± 0.13	0.27 ± 0.12	0.02 (−0.07 to 0.10)	0.685	0.35 ± 0.13	0.25 ± 0.10	0.10 (0.01 to 0.18)	**0** **.** **029**
Nasal	0.30 ± 0.11	0.26 ± 0.11	0.04 (−0.03 to 0.12)	0.248	0.34 ± 0.14	0.24 ± 0.09	0.11 (0.02 to 0.19)	**0** **.** **015**
CSI								
Fovea	0.74 ± 0.09	0.64 ± 0.06	0.10 (0.05 to 0.15)	**<0** **.** **001**	0.66 ± 0.06	0.65 ± 0.09	0.01 (−0.05 to 0.06)	0.807
Superior	0.68 ± 0.11	0.64 ± 0.08	0.04 (−0.02 to 0.11)	0.188	0.62 ± 0.07	0.65 ± 0.12	−0.03 (−0.10 to 0.04)	0.454
Temporal	0.70 ± 0.09	0.66 ± 0.07	0.04 (−0.02 to 0.09)	0.182	0.65 ± 0.07	0.67 ± 0.10	−0.02 (−0.08 to 0.04)	0.459
Inferior	0.67 ± 0.10	0.65 ± 0.06	0.02 (−0.04 to 0.08)	0.407	0.62 ± 0.06	0.67 ± 0.10	−0.04 (−0.10 to 0.01)	0.113
Nasal	0.70 ± 0.10	0.63 ± 0.07	0.05 (−0.01 to 0.10)	0.098	0.62 ± 0.06	0.64 ± 0.11	−0.02 (−0.09 to 0.04)	0.449
CVV (mm^3^)								
Fovea	0.07 ± 0.03	0.08 ± 0.04	−0.01 (−0.03 to 0.01)	0.408	0.10 ± 0.04	0.07 ± 0.03	0.03 (0.01 to 0.05)	**0** **.** **043**
Superior	0.15 ± 0.07	0.15 ± 0.06	−0.00 (−0.05 to 0.04)	0.828	0.21 ± 0.09	0.15 ± 0.07	0.07 (0.01 to 0.12)	**0** **.** **019**
Temporal	0.15 ± 0.06	0.15 ± 0.06	0.00 (−0.04 to 0.04)	0.852	0.20 ± 0.08	0.14 ± 0.05	0.06 (0.02 to 0.11)	**0** **.** **010**
Inferior	0.15 ± 0.06	0.15 ± 0.07	0.00 (−0.04 to 0.05)	0.932	0.21 ± 0.09	0.14 ± 0.07	0.07 (0.01 to 0.12)	**0** **.** **018**
Nasal	0.16 ± 0.08	0.15 ± 0.07	0.01 (−0.04 to 0.06)	0.825	0.21 ± 0.08	0.15 ± 0.08	0.06 (0.01 to 0.11)	**0** **.** **021**
CVI								
Fovea	0.23 ± 0.09	0.36 ± 0.06	−0.13 (−0.19 to −0.07)	**<0** **.** **001**	0.31 ± 0.07	0.36 ± 0.10	−0.04 (−0.10 to 0.03)	0.132
Superior	0.30 ± 0.12	0.36 ± 0.08	−0.07 (−0.14 to −0.00)	**0** **.** **046**	0.35 ± 0.08	0.36 ± 0.14	−0.01 (−0.10 to 0.06)	0.714
Temporal	0.29 ± 0.10	0.34 ± 0.07	−0.05 (−0.11 to 0.01)	0.125	0.32 ± 0.07	0.34 ± 0.10	−0.02 (−0.07 to 0.04)	0.570
Inferior	0.32 ± 0.11	0.35 ± 0.06	−0.04 (−0.10 to 0.03)	0.261	0.33 ± 0.08	0.34 ± 0.09	−0.01 (−0.08 to 0.05)	0.657
Nasal	0.30 ± 0.11	0.37 ± 0.08	−0.07 (−0.13 to −0.01)	**0** **.** **035**	0.36 ± 0.07	0.38 ± 0.11	−0.02 (−0.09 to 0.04)	0.494

SFCT, subfoveal choroidal thickness; CSV, choroidal stromal volume; CSI, choroidal stromal index; CVV, choroidal vasculary volume; CVI, choroidal vascularity index. Significant *P* values are in bold.

a Independent-sample *t*-test.

#### 3.4.2 Choroidal stromal volume (CSV)

In CRVO patients, CSV values were similar between SRD and non-SRD eyes across all macular subfields (*P* > 0.05) (see Table 5).

In BRVO patients, CSV was significantly higher in SRD eyes across all regions (fovea: 0.19 ± 0.07 mm^3^; superior: 0.36 ± 0.14 mm^3^; temporal: 0.37 ± 0.14 mm^3^; inferior: 0.35 ± 0.13 mm^3^; nasal: 0.34 ± 0.14 mm^3^) than in non-SRD eyes (fovea: 0.13 ± 0.06 mm^3^; superior: 0.26 ± 0.11 mm^3^; temporal: 0.28 ± 0.10 mm^3^; inferior: 0.25 ± 0.10 mm^3^; nasal: 0.24 ± 0.09 mm^3^) (*P* < 0.05) (see Table 5).

#### 3.4.3 Choroidal stromal index (CSI)

In CRVO patients, the only CSI difference was observed in the foveal region, where SRD eyes (0.74 ± 0.09) had higher values than non-SRD eyes (0.64 ± 0.06) (MD, 0.10; 95% CI, 0.05–0.15; *P* < 0.001). No statistically significant differences were observed between the two groups in the remaining subfields (*P* > 0.05) (see Table 5).

In BRVO patients, CSI did not differ significantly between groups in any region (*P* > 0.05) (see Table 5).

#### 3.4.4 Choroidal vascular volume (CVV)

CVV was elevated in BRVO eyes with SRD across all five macular regions. Foveal, superior, temporal, inferior, and nasal CVV values were 0.10 ± 0.04 mm^3^, 0.21 ± 0.09 mm^3^, 0.20 ± 0.08 mm^3^, 0.21 ± 0.09 mm^3^, and 0.21 ± 0.08 mm^3^ in the SRD group, compared with 0.07 ± 0.03 mm^3^, 0.15 ± 0.07 mm^3^, 0.14 ± 0.05 mm^3^, 0.14 ± 0.07 mm^3^, and 0.15 ± 0.08 mm^3^ in the non-SRD group (*P* < 0.05). No CVV region reached statistical significance in CRVO (*P* > 0.05) (see Table 5).

#### 3.4.5 Choroidal vascularity index (CVI)

In CRVO patients, CVI was reduced in SRD eyes in the foveal (0.23 ± 0.09), superior (0.30 ± 0.12), and nasal regions (0.30 ± 0.11) relative to non-SRD eyes (foveal: 0.36 ± 0.06; superior: 0.36 ± 0.08; nasal: 0.37 ± 0.08) (*P* < 0.05). Temporal and inferior CVI values were not significantly different (*P* > 0.05) (see Table 5).

In BRVO, CVI remained comparable between SRD and non-SRD eyes in all analyzed regions (*P* > 0.05) (see Table 5).

### 3.5 Representative UWF-SS-OCTA images

Representative UWF-SS-OCTA images are shown to illustrate the imaging characteristics associated with SRD status and RVO subtype. Figures 5, 6 show representative CRVO and BRVO eyes with retinal capillary plexus density maps and choroidal parameter maps, whereas Figures 7, 8 show CRVO-ME eyes with and without SRD, respectively.

**Figure 5:**
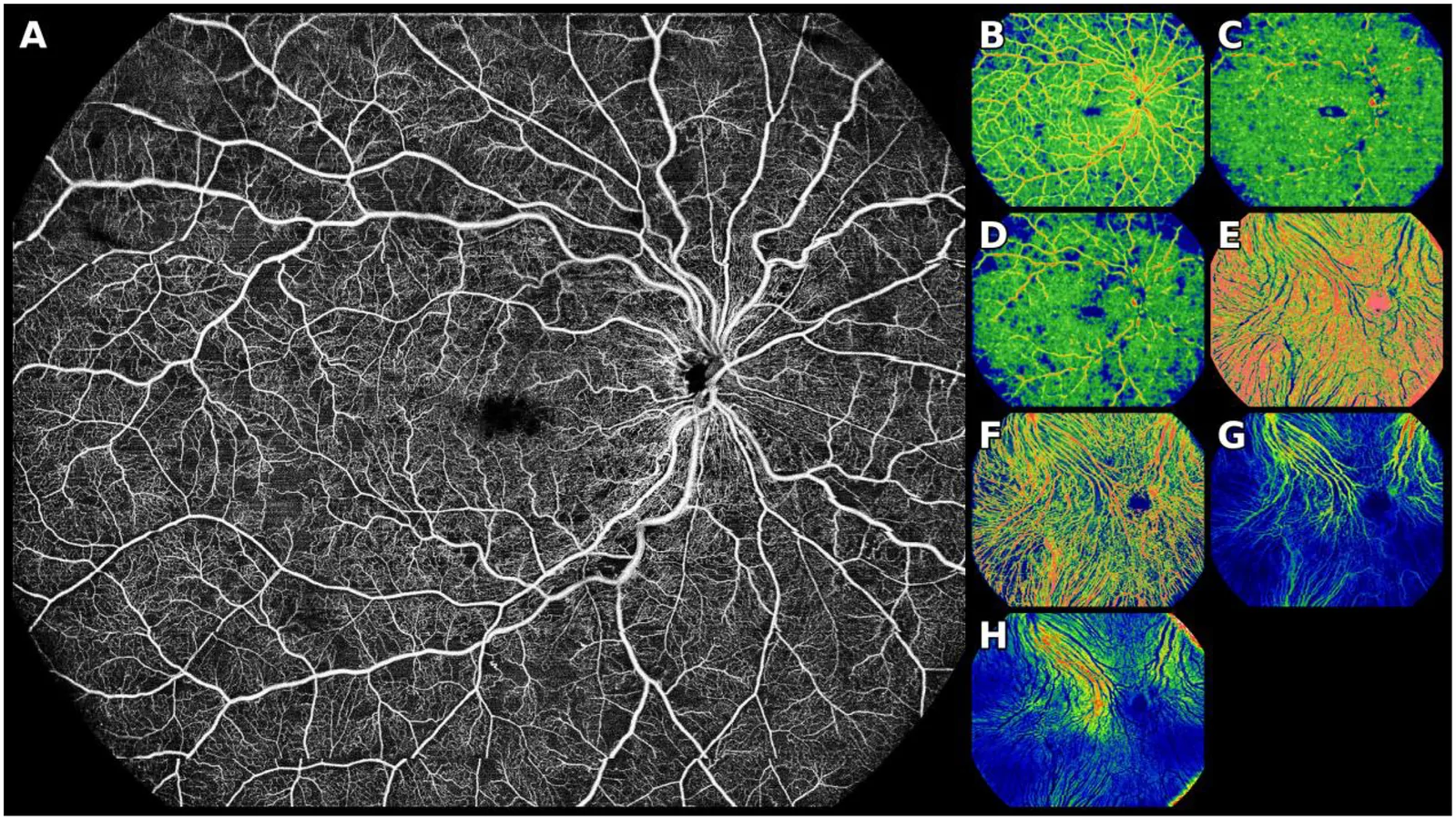
Representative UWF-SS-OCTA images of an eye with central retinal vein occlusion (CRVO). **(A)** En face angiogram of the full-retinal slab. **(B–D)** Color-coded flow density maps of the superficial capillary plexus (SCP), middle capillary plexus (MCP), and deep capillary plexus (DCP), respectively. **(E–H)** Three-dimensional choroidal parameter maps: choroidal stromal index (CSI), choroidal vascularity index (CVI), choroidal vascular volume (CVV), and choroidal stromal volume (CSV). Scan range: 24 mm × 20 mm.

**Figure 6:**
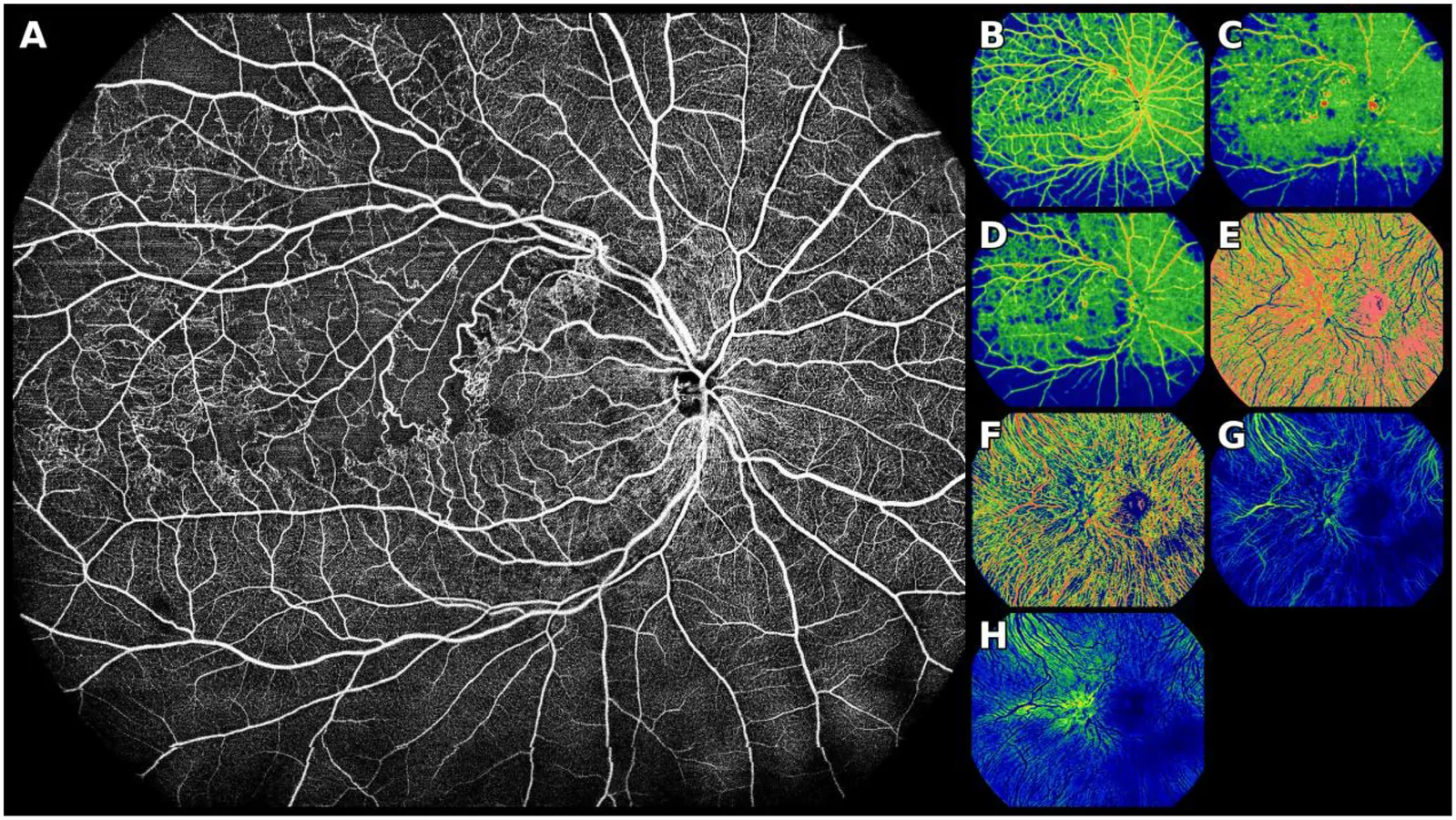
Representative UWF-SS-OCTA images of an eye with branch retinal vein occlusion (BRVO). **(A)** En face angiogram of the full-retinal slab. **(B–D)** Color-coded flow density maps of the superficial capillary plexus (SCP), middle capillary plexus (MCP), and deep capillary plexus (DCP), respectively. **(E–H)** Three-dimensional choroidal parameter maps: choroidal stromal index (CSI), choroidal vascularity index (CVI), choroidal vascular volume (CVV), and choroidal stromal volume (CSV). Scan range: 24 mm × 20 mm.

**Figure 7:**
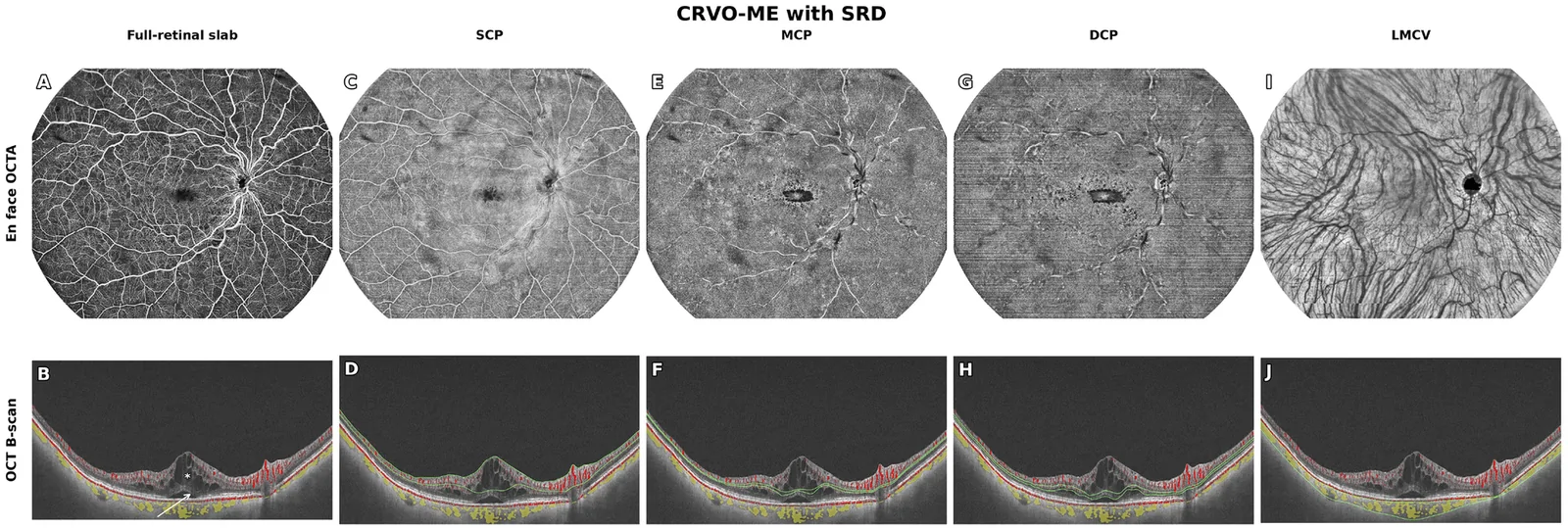
Representative UWF-SS-OCTA images of a CRVO-ME eye with serous retinal detachment (SRD). **(A)** Full-retinal slab en face OCTA image. **(B)** Cross-sectional B-scan with flow overlay (red: retinal flow; yellow: choroidal flow) showing a peaked fovea with large intraretinal cystoid spaces (asterisk) and a hyporeflective subretinal space (arrow) between the elevated neurosensory retina and the retinal pigment epithelium. **(C,E,G,I)** En face OCTA images of the superficial capillary plexus (SCP), middle capillary plexus (MCP), deep capillary plexus (DCP), and the large and medium choroidal vessels (LMCV), respectively. **(D,F,H,J)** Corresponding B-scans with green segmentation lines delineating each slab. Scan range: 24 mm × 20 mm.

**Figure 8:**
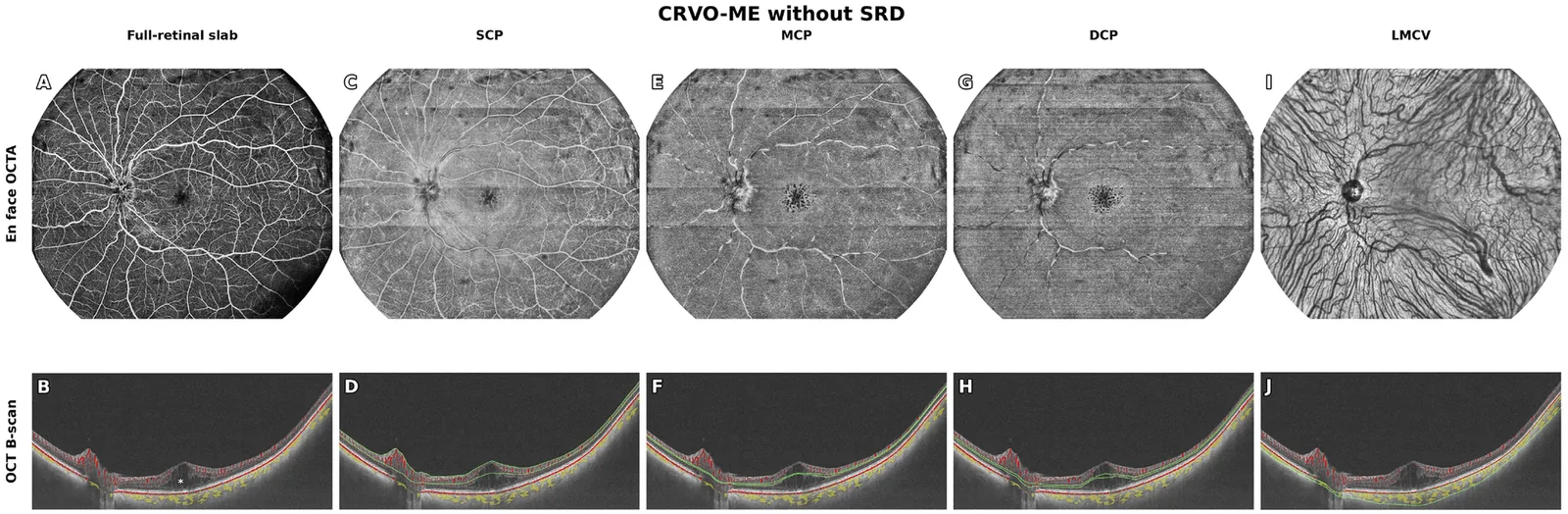
Representative UWF-SS-OCTA images of a CRVO-ME eye without SRD. **(A)** Full-retinal slab en face OCTA image. **(B)** Cross-sectional B-scan showing intraretinal cystoid edema (asterisk), with the neurosensory retina attached to the retinal pigment epithelium and no subretinal fluid. **(C,E,G,I)** En face OCTA images of the superficial capillary plexus (SCP), middle capillary plexus (MCP), deep capillary plexus (DCP), and the large and medium choroidal vessels (LMCV), respectively. **(D,F,H,J)** Corresponding B-scans with green segmentation lines delineating each slab. Scan range: 24 mm × 20 mm.

## 4 Discussion

This retrospective study used UWF-SS-OCTA to compare retinal and choroidal features between treatment-naive RVO-ME eyes with and without SRD. The results showed that SRD eyes in both CRVO and BRVO exhibited significantly greater CMT, lower FAZ-CI and shorter disease duration than non-SRD eyes in both RVO subtypes; in addition, the proportion of ischemic eyes was higher in the CRVO-SRD group, and in BRVO the extent of the occluded retinal area was greater in SRD eyes, whereas the distribution of occlusion sites did not differ significantly, with the superotemporal branch being the most commonly affected. Regarding retinal microcirculation, the MCP-VFD was reduced across all macular subfields in CRVO, whereas BRVO eyes with SRD showed a more diffuse multilayer VFD reduction, with lower SCP-VFD in all macular subfields and lower MCP-VFD and DCP-VFD in four of five subfields. The two RVO subtypes showed distinct choroidal features: in the BRVO-SRD group, SFCT was significantly increased, while CSV and CVV were elevated in all macular sectors; in the CRVO-SRD group, CSI was increased in the central foveal region, whereas CVI was decreased in the central foveal, superior, and nasal sectors. Taken together, RVO-ME eyes with SRD showed greater macular thickening, more irregular FAZ morphology, broader MCP-predominant capillary rarefaction, and subtype-specific choroidal alterations.

CMT is a classical morphological indicator for evaluating the severity of RVO-ME. We found that CMT was significantly higher in the SRD group than in the non-SRD group in both CRVO and BRVO patients (Figure 1), indicating that SRD is closely associated with more severe macular edema. This finding is consistent with the structural features of SRD secondary to RVO-ME reported by Liu et al. (19). Macular edema is essentially caused by vascular leakage and tissue fluid accumulation resulting from impaired retinal venous outflow and disruption of the blood-retinal barrier. The presence of SRD is generally considered to indicate that fluid leakage has exceeded the outer retinal barrier, leading to separation between the RPE and the neurosensory retina, and ultimately resulting in a marked increase in macular structural thickness (2, 20).

Furthermore, patients in the CRVO-SRD group had significantly worse baseline BCVA and a higher proportion of ischemic eyes than those in the non-SRD group, whereas no such differences were observed in BRVO. This finding may be related to the more severe ischemic injury and greater involvement of the macular fovea in CRVO itself (13). Notably, eyes with SRD had significantly shorter disease duration in both subtypes, possibly because the accompanying severe macular thickening and outer blood–retinal barrier disruption (19) may lead to sudden visual decline, prompting earlier presentation and shortening the documented interval from symptom onset to initial imaging. Consistently, CRVO eyes with SRD had significantly worse BCVA than those without. In BRVO, although BCVA did not differ significantly between groups, CMT was significantly higher in eyes with SRD; given that visual disturbances such as metamorphopsia in RVO-related macular edema correlate with retinal thickening and SRD extent and may be underestimated by letter acuity (21), severe macular edema may likewise prompt earlier presentation.

We quantitatively analyzed the morphological parameters of the FAZ. The results showed no significant differences in FAZ area, perimeter, or FD-300 between the two groups, whereas FAZ-CI was significantly reduced (Figure 2), suggesting that FAZ morphology was more irregular in eyes with SRD. CI is an index reflecting the geometric shape of the FAZ. The normal FAZ is approximately circular, whereas its contour becomes irregular under ischemic or inflammatory conditions (22). Previous FAZ-related studies have commonly used area and perimeter as classical morphological indicators for assessing macular microcirculatory ischemia (22–24). Fan et al. (24) found that some patients with RVO complicated by macular edema did not show obvious FAZ enlargement, but already had occult microvascular disorganization. Chen et al. (25) also suggested that FAZ area alone cannot comprehensively evaluate retinal microvascular damage in RVO. Our findings are consistent with these conclusions. We speculate that the mechanical compression of retinal tissue caused by macular edema may partly offset the tendency toward FAZ enlargement induced by retinal ischemia. This may explain why no intergroup differences in FAZ area or perimeter were observed in patients with RVO-ME complicated by SRD, although this inference requires further validation in longitudinal studies. Meanwhile, FD-300 is a classical quantitative indicator of superficial capillary perfusion in the annular region surrounding the FAZ (26). The absence of a significant intergroup difference in FD-300 in this study further suggests that SRD was not associated with a marked reduction in superficial perfusion in this region. These findings suggest that SRD-related microcirculatory injury is mainly manifested as distortion of the microvascular contour rather than a global decrease in perfusion density, representing two distinct dimensions of pathological damage.

Fan et al. (24) proposed that FAZ morphological parameters, such as CI, may be more sensitive than area-based parameters and may serve as important indicators for evaluating subtle macular structural damage. Zhang et al. (22) suggested that CI can effectively reflect the degree of macular microvascular architectural remodeling. In this study, FAZ-CI was significantly reduced in the SRD group, suggesting that perimacular capillary nonperfusion and blood flow redistribution in eyes with SRD may disrupt the contour of the FAZ boundary and its normal circular configuration without altering the overall extent of the FAZ. Vujosevic et al. (27) also found that abnormal FAZ morphology was closely associated with outer blood-retinal barrier damage and serous retinal detachment. In healthy populations, FAZ-CI is generally maintained at 0.85–0.95 (28). In the present study, CI in the SRD group clearly deviated from the normal reference range. We therefore believe that, compared with FAZ area, perimeter, and FD-300, FAZ-CI may be a sensitive morphological biomarker for identifying occult macular microcirculatory injury in patients with RVO-ME complicated by SRD, although its clinical utility remains to be established.

The retinal SCP, MCP, and DCP are core structures that maintain macular microcirculatory homeostasis. Perfusion abnormalities in these three vascular plexuses are an important pathological basis for the development and progression of RVO-ME (16). In BRVO patients, VFD in almost all macular sectors of the SCP and MCP and in most sectors of the DCP was significantly lower in the SRD group than in the non-SRD group. In contrast, the changes in the CRVO-SRD group showed regional and layer-specific selectivity. For example, in CRVO patients, MCP-VFD was significantly reduced in all sectors in the SRD group, whereas significant reductions in VFD were observed only in the nasal SCP and inferior DCP. This difference may reflect variations between CRVO and BRVO in the site of occlusion, compensatory capacity of collateral circulation, and severity of ischemia (29, 30).

Notably, MCP showed the most consistent decrease in VFD in patients with both CRVO-SRD and BRVO-SRD. MCP-VFD was significantly reduced in all sectors in CRVO-SRD (Figure 3) and in all sectors except the inferior sector in BRVO-SRD (Figure 4), a feature with important pathophysiological relevance. Independent analyses of the MCP in RVO remain relatively limited, as previous OCTA studies of RVO have mainly focused on the SCP and DCP (10, 23–26, 30). In the present study, the MCP was quantitatively analyzed as an independent vascular layer. Anatomical and OCTA studies have shown that the MCP is an independent vascular layer located between the inner plexiform layer and the inner nuclear layer. Anatomically, it connects the SCP and DCP and participates in the regulation of interlaminar macular blood flow (31, 32). Park et al. (32) were the first to systematically characterize the anatomical features of the MCP in healthy and diabetic human eyes using OCTA projection-resolved technology, confirming its high sensitivity to ischemic and metabolic stress. Onishi et al. (31) also reported that the MCP was significantly associated with the severity of diabetic retinopathy and paralleled pathological changes in the DCP, suggesting that the MCP may play a key role in retinal microcirculatory disturbance. Using high-resolution OCTA, Nesper (33) further confirmed that the MCP plays an important role in constructing the parafoveal capillary network and maintaining outer retinal perfusion. These studies indicate that the MCP has important functions in retinal microcirculation and is highly sensitive to ischemia. Similar roles may also exist in RVO, but they have not yet been directly confirmed. The consistent decrease in MCP-VFD observed in this study suggests that MCP-VFD may be a valuable OCTA parameter for detecting microvascular damage associated with RVO-ME complicated by SRD. However, whether MCP damage occurs before SRD or represents a secondary change after SRD formation remains to be confirmed in longitudinal studies.

As the core blood supply structure of the deep retina, the DCP primarily supports nutritional perfusion of the outer retina. Previous studies have shown that DCP nonperfusion may reflect more severe microvascular damage and is associated with an increased risk of retinal complications and long-term prognosis (34, 35). In RVO, ischemia, inflammatory factors, and VEGF-mediated increases in vascular permeability are considered to participate in the formation of macular edema and SRD (4, 19, 36). In this study, DCP-VFD was significantly reduced in all quadrants except the foveal sector in the BRVO-SRD group, whereas only selective hypoperfusion in the inferior sector was observed in the CRVO-SRD group. Based on OCTA, Nesper et al. (33) demonstrated that the parafoveal deep capillary network has complex interlaminar connections and relatively independent supply and drainage structures. This suggests that different macular sectors may show incompletely consistent perfusion patterns when venous outflow is impaired. However, direct evidence is still lacking to prove that this anatomical feature can fully explain the regional selectivity of DCP injury in RVO-ME complicated by SRD. This difference may be related to the individual extent of occlusion, anatomical variations in the macular vascular arcade, the degree of collateral circulation formation, the method used for SRD grouping, the influence of macular edema on deep OCTA signals, and sample size. Further studies are needed to validate this finding. Clinically, a decrease in DCP-VFD in patients with RVO-ME complicated by SRD, especially a multiquadrant decrease in BRVO, should suggest the possibility of more severe deep capillary perfusion impairment. This finding may be further interpreted together with fundus fluorescein angiography nonperfusion areas, outer retinal integrity, and longitudinal changes in visual acuity.

For BRVO, we further compared the location and extent of retinal areas affected by occlusion between eyes with and without SRD. In both groups, the occluded branch was most frequently the superotemporal branch, with no significant difference in the distribution of occlusion locations; this finding is consistent with the established observation that BRVO preferentially occurs at superotemporal arteriovenous crossings, where the crossing anatomy and local hemodynamic conditions predispose the vein to compression (37, 38). In contrast, the extent of the occluded retinal area was significantly greater in eyes with SRD. A more extensive occlusion exposes a larger retinal area to elevated venous pressure and hypoxia, which may increase the production of VEGF and inflammatory mediators (19, 36) and thereby promote extensive leakage beyond the compensatory capacity of the outer blood-retinal barrier. Moreover, intraretinal fluid may migrate from a distant occlusion site to the subfoveal space (39), which may explain why SRD can still occur when the occlusion is extramacular, provided that the affected area is extensive. These findings suggest that, beyond the mere presence of occlusion, the spatial burden of the occluded area may be a factor associated with SRD development in BRVO.

This study further analyzed differences in choroidal structural and perfusion parameters between RVO-ME eyes with and without SRD. These parameters are clinically relevant because the choroid supports the RPE and outer retina, participates in fluid exchange across the outer blood-retinal barrier (5), and may be affected by hydrostatic, inflammatory, and VEGF-mediated changes after retinal venous occlusion (6). The results showed no significant difference in SFCT between CRVO eyes with and without SRD, whereas SFCT was significantly increased in the BRVO-SRD group. Current findings regarding SFCT in patients with RVO remain controversial (40). Zhao et al. (10) found occult choroidal microcirculatory abnormalities in both RVO eyes and contralateral fellow eyes, suggesting that SFCT alone is insufficient to comprehensively reflect choroidal pathological status. The significantly increased SFCT in the BRVO-SRD group suggests that, in focal venous occlusion, SFCT thickening accompanies the presence of SRD and may reflect baseline choroidal congestion or edema. Although SFCT tended to increase in the CRVO-SRD group in this study, the difference did not reach statistical significance, possibly because of the sample size and interindividual variability.

Compared with SFCT as a single thickness parameter, three-dimensional choroidal parameters can provide layer-specific quantitative information on choroidal vascular and stromal components. CVV and CSV quantify choroidal vascular volume and choroidal stromal volume, respectively, whereas CVI and CSI describe the relative proportions of vascular and stromal components (41–43). In this study, CVV and CSV were significantly increased in all macular sectors in the BRVO-SRD group, whereas CVI and CSI remained comparable between BRVO-SRD and non-SRD eyes. This finding indicates that vascular and stromal components may expand synchronously rather than showing a selective increase in the proportion of one component within the total choroidal volume. This pattern is consistent with the focal venous occlusion characteristics of BRVO. Impaired local venous outflow may increase hydrostatic pressure and enhance choroidal vascular permeability through VEGF and inflammatory mediators, thereby inducing both vascular luminal dilation and interstitial fluid retention (4, 8, 41, 44). From a pathophysiological perspective, the choroid is the principal blood supply to the outer retina, and increased vascular permeability and volume expansion may increase the burden of outward fluid drainage by the RPE, concomitant with decompensation of the outer blood-retinal barrier and subretinal fluid accumulation (45, 46). Accordingly, the increased choroidal volume burden in BRVO-SRD may result from local venous congestion and may also contribute to the development and persistence of SRD through the mechanisms described above; the temporal relationship between these processes requires clarification in longitudinal studies.

In contrast, CRVO-SRD did not show pan-sector increases in CVV or CSV, nor did it show significant SFCT thickening. Foveal CSI increased in CRVO-SRD, whereas CVI decreased in selected sectors. This finding suggests that the choroidal differences primarily involved the relative composition of the vascular and stromal components rather than an expansion of overall choroidal volume. CRVO involves a broader retinal venous drainage system, and the burdens of ischemia, inflammation, and capillary hypoperfusion are usually greater than those in BRVO (36, 47). In this context, the relative decrease in choroidal vascular components may reflect reduced perfusion, compression of vascular lumens by stromal edema, or atrophy of vascular components after chronic ischemia. This interpretation is also consistent with previous studies suggesting that CVI may reflect choroidal abnormalities in RVO more sensitively than SFCT (7, 43). By simultaneously quantifying the absolute volumes and relative proportions of the choroidal vascular and stromal components using UWF-SS-OCTA, we found that the patterns of choroidal alterations accompanying CRVO-SRD and BRVO-SRD differed, suggesting that they may not simply represent different degrees of the same choroidal response.

Taken together, these multidimensional retinal and choroidal imaging findings suggest that eyes with RVO-ME and SRD should not be assumed to have the same disease course or treatment response as eyes without SRD. Previous studies have shown that choroidal parameters are dynamic and treatment-responsive: SFCT decreases after anti-VEGF therapy for RVO (48), choroidal volume and stromal area decrease after intravitreal anti-VEGF treatment (11, 44, 49), and CVI changes over the course of BRVO-associated macular edema (12, 13). Baseline choroidal metrics are also associated with treatment outcomes, as exemplified by the reported association between baseline SFCT and the response to intravitreal bevacizumab (50). Retinal OCTA parameters (including macular thickness, FAZ metrics, and vascular density) have likewise been shown to change dynamically after anti-VEGF treatment (26, 51). These longitudinal observations raise the possibility that the baseline differences in parameters between eyes with and without SRD that we identified may carry prognostic information.

In clinical assessment, integrating SRD status with CMT, FAZ morphology, layer-specific retinal VFD, ischemic status, extent of occlusion, and three-dimensional choroidal parameters may facilitate a more comprehensive imaging evaluation and more complete characterization of eyes with extensive structural and microcirculatory involvement at initial presentation. Specifically, BRVO-SRD was characterized by diffuse reductions in retinal VFD accompanied by increases in SFCT, CVV, and CSV, suggesting more pronounced retinal microcirculatory involvement and choroidal structural expansion; CRVO-SRD, in contrast, was characterized predominantly by widespread MCP-VFD reduction accompanied by alterations in choroidal composition, consistent with a more severe microcirculatory injury phenotype. The distinction between these subtypes suggests that RVO subtype and SRD status should be treated as stratification dimensions rather than considering RVO-ME a homogeneous population. This study cannot yet establish a treatment algorithm, but it provides a reasonable imaging-based stratification framework for future prospective studies evaluating anti-VEGF treatment response, recurrence, and long-term visual outcomes.

Several limitations should be acknowledged. First, this was a retrospective, single-center study with a relatively small sample size, which may have limited the statistical power and generalizability of the findings. Second, the cross-sectional design permits only associations between SRD and retinal or choroidal alterations to be inferred; causal and temporal relationships cannot be determined, and the prognostic value of these biomarkers requires validation in longitudinal studies. Third, multiple comparisons were performed without formal adjustment; these analyses should be regarded as exploratory, and we therefore reported 95% confidence intervals alongside *P* values. Finally, although key clinical variables were collected, residual confounding remains possible: axial length was not measured, nonperfusion extent on FFA was not quantified, and disease duration differed significantly between groups, such that some imaging differences may partly reflect the disease phase rather than SRD itself.

## 5 Conclusions

In treatment-naive RVO-ME eyes evaluated by UWF-SS-OCTA, SRD was associated with greater macular thickening, more irregular FAZ morphology, and more pronounced retinal microcirculatory impairment, with MCP-VFD reduction as the most consistent feature. Choroidal volume expansion in BRVO and compositional remodeling in CRVO suggest subtype-specific choroidal involvement; together, these findings support the potential value of layer-specific retinal perfusion metrics and three-dimensional choroidal parameters for imaging-based stratification.

## Data Availability

The raw data supporting the conclusions of this article will be made available by the authors, without undue reservation.
